# Lightguide–Lens Array Side–Absorption Concentrated Photovoltaic (CPV) System with an Extended Acceptance Angle

**DOI:** 10.1021/acsomega.6c05654

**Published:** 2026-10-28

**Authors:** An-Chi Wei, Yi-Hung Hsu, Jyh-Rou Sze

**Affiliations:** † Graduate Institute of Energy Engineering, 34911 National Central University, Taoyuan City 320317, Taiwan, R.O.C; ‡ Department of Mechanical Engineering, National Central University, Zhongli District, Taoyuan City 320317, Taiwan, R.O.C; § National Center for Instrumentation Research, National Institutes of Applied Research, Hsinchu City 300092, Taiwan, R.O.C

## Abstract

A lightguide–lens–array side-absorption concentrated photovoltaic system (LLA-SCPV) is proposed and experimentally demonstrated for wide-angle operation. The bottom surface of the lightguide is patterned with reflective V-grooves to direct incident light toward solar cells mounted on its sidewalls. Both the lightguide and the lens array are made of PMMA. The device exhibits a simulated acceptance angle of at least ±12° and an experimental acceptance angle of ±15°. Through an optical efficiency breakdown, manufacturing profile deviations and reduced reflectance of the V-groove facets are identified as the primary factors of the optical loss, while the V-groove profile deviations broaden the angular response. For further performance improvement, simulations under realistic industrial conditions indicate that replacing PMMA with fused silica, increasing V-groove reflectance to 0.86 and reducing the corner radius to 0.1 mm can enhance the optical efficiency to 67.7% while maintaining an acceptance angle of approximately ±15.5°.

## Graphical abstract

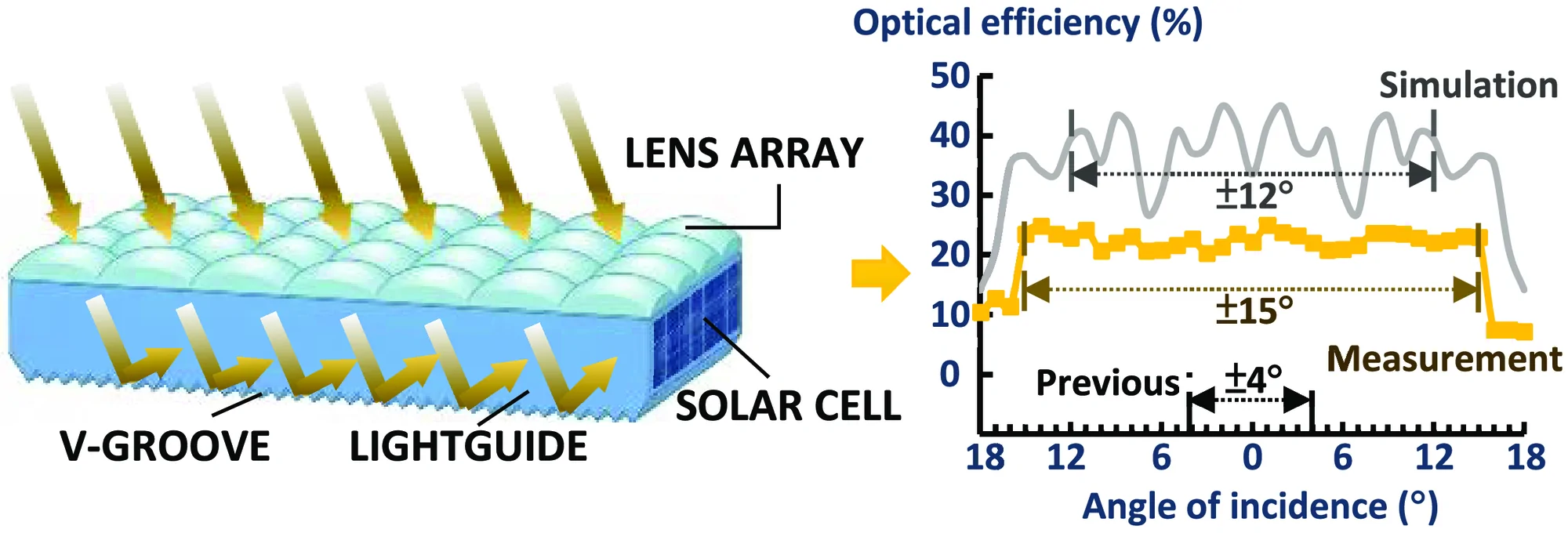

## 1 Introduction

With the increasing energy demand, solar energy is regarded as one of the most promising energy sources in recent decades. Among the solar energy technologies, photovoltaics (PV) provide a direct approach to converting solar energy into electricity, and the PV market is dominated by crystalline-silicon modules because of their mature manufacturing. The efficiency of crystalline-silicon cells has reached 27.6% (concentration) in the laboratory, while commercial crystalline-silicon modules typically achieve efficiencies of over 20%.^1^ Continued advances in photovoltaic technology rely on innovation spanning materials and devices, system design, and module integration. At the material and device levels, advances include approaches such as III–V semiconductors, perovskite/Si tandems, tunnel oxide passivated contact (TopCon), and heterojunction (HJT) architectures,^2–5^ as well as emerging interface- and band-engineering strategies which have demonstrated promising results in photovoltaic and other advanced optoelectronic devices.^6–10^ At the system level, various approaches have been explored, such as heat-management strategies involving photovoltaic/thermal (PV/T), photovoltaic-thermoelectric generator (PV-TEG), and microchannel systems.^11–13^ At the module and system-integration levels, ongoing developments include novel configurations such as building-integrated photovoltaics (BIPV), bifacial solar modules, space-grade concentrators.^14–16^ In space applications, the concentrated photovoltaic (CPV) system is an appropriate system to power satellites because of its high efficiency and elimination of the issue of cleanness for optics in space.^17^ CPV systems can be categorized into three types based on concentration ratio: low concentration (LCPV), medium concentration (MCPV), high concentration (HCPV). The concentration ratios of LCPV, MCPV, and HCPV are 1–40x, 40–300x, and 300–2000x, respectively.^18^ HCPV systems, generally utilize III–V cells to achieve efficiencies of around 40%, provide high power density and are suitable for applications such as utility-scale power generation and space photovoltaics. However, their high concentration ratios generally result in narrow acceptance angles, necessitating precise and power-intensive tracking and increasing the burden of tracker maintenance.^19,20^ Extending the acceptance angle, such as using LCPV systems, mitigates this issue by reducing tracking accuracy requirements and tracking activity and enabling partial concentration of diffuse radiation, thereby improving off-axis performance.^21,22^ Generally, LCPV systems, with larger acceptance angles and lower tracking requirements, are attractive for rooftop PV and BIPV applications requiring low tracking load, simple system, and low maintenance costs. CPVs can also be broadly categorized into two configurations: vertical concentration and side concentration. In vertical-concentration designs, large acceptance angles exceeding ±40° have been achieved using III–V/Si assembled PVs, compound parabolic concentrators (CPCs), tracking-integrated CPVs, and microtracking techniques.^23–26^ For side-concentration configurations, self-tracking mechanisms utilizing thermally generated features, such as thermally induced apertures and thermally formed reflectors, have demonstrated acceptance angles ranging from ±16° to ±25°.^27,28^ However, these thermally assisted methods may suffer from heat-related issues. Alternatively, an optically assisted method realizes side concentration using optical components, thereby minimizing thermal risks under sunlight. Nevertheless, the optically assisted method demonstrated a limited acceptance angle of about ±4.4° when implemented with a V-groove array.^19^ Therefore, further improvement of the acceptance angle is required. The aforementioned categories of CPV techniques are summarized and illustrated in Figure 1.

**Figure 1:**
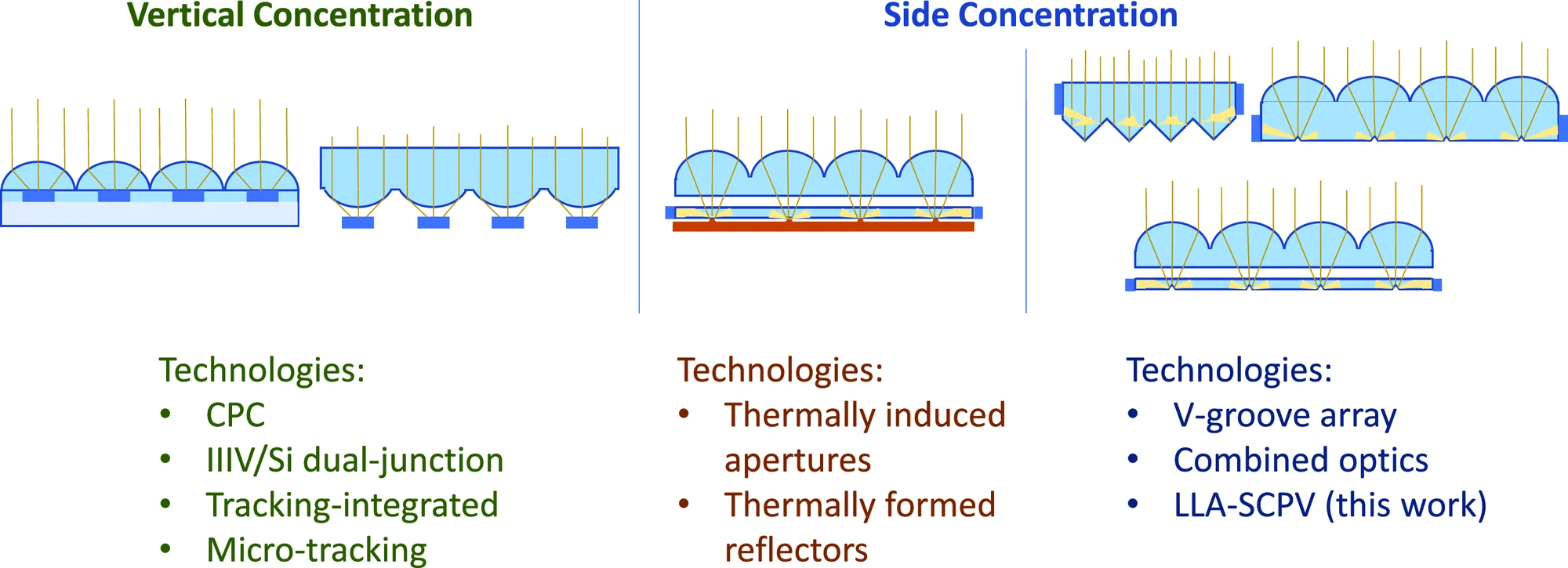
Classification of concentrated photovoltaic systems (CPVs). The vertical-concentration CPVs, concentrating sunlight vertically onto the solar cells (blue rectangles), whereas the side-concentration CPVs guide sunlight toward solar cells on the sides of the lightguide.

To further extend the acceptance angle of the optically assisted method, we herein propose a lightguide–lens–array side-absorption CPV (LLA-SCPV) that integrates a V-groove lightguide with a lens array. The proposed LLA-SCPV is evaluated through optical analysis to demonstrate its capability for acceptance-angle enhancement while maintaining low thermal risk. In the following sections, the design, fabrication, and performance of the proposed system are presented and discussed in detail.

## 2 Design and Modeling

### 2.1 System Design

The proposed LLA-SCPV consists of a lens array and a lightguide for collecting and guiding sunlight, respectively. The solar cells are placed on the sides of the lightguide, as illustrated in Figure 2. Since the lens array is the first element along the optical path, it has to possess a sufficiently large acceptance angle to transmit solar energy to the subsequent elements. The lightguide is positioned below the lens array, separated by an air gap, and features optimized V-grooves to provide high light-guiding efficiency. In this work, a demonstration is presented using a lens array and a lightguide, both made of poly­(methyl methacrylate) (PMMA), with the lightguide coated with aluminum.

**Figure 2:**
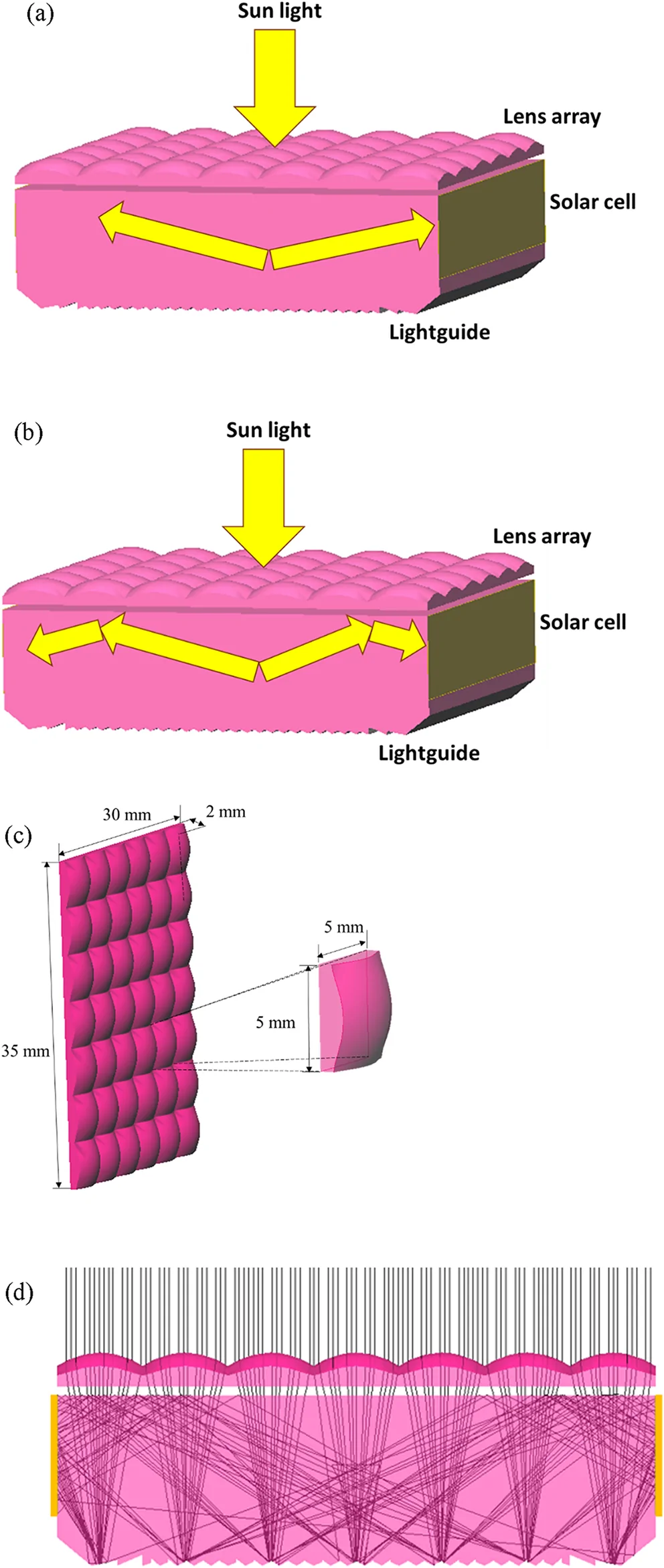
Structure of the proposed LLA-SCPV (a) system design with a direct-aiming mechanism, (b) indirect-aiming mechanism, (c) dimensions of components, and (d) ray-tracing plot using normally incident rays. (a), (b) and (d) are adapted with permission from ref 29 whereas (c) is reprinted with permission from ref 29

### 2.2 Ray-Tracing Model

The light-coupling behavior of the proposed concentrator is governed by two mechanisms: direct-aiming and indirect-aiming.^19^ In the direct-aiming mechanism, rays focused by the lens array impinge on the V-groove and are reflected directly to the side-mounted solar cells. In the indirect-aiming mechanism, rays first reflect to the top surface of the lightguide, where they undergo total internal reflection (TIR) before reaching the solar cells. Based on the geometric ray tracing, the design references the refractive index of PMMA (*n* ≈ 1.4936 at 550 nm) to evaluate the TIR condition and to determine the feasible V-groove-tilt range (∼21°–45°) that supports these TIR-assisted paths. By combining single-bounce reflection and TIR-assisted routing, these two mechanisms jointly contribute to the broadened acceptance angle achieved in this work.

To evaluate the optical performance of the proposed LLA-SCPV system, we constructed a three-dimensional ray-tracing model in LightTools. The PMMA lens array consisted of aspheric plano-convex lenslets arranged over an overall area of 30 mm × 35 mm with a lens pitch of 5 mm. The PMMA lightguide was mounted below the lens array, and its V-grooves serve as the reflecting surface. The optimized air gap of 2 mm was set between the bottom surface of the lens array and the top surface of the lightguide. In this study, the acceptance angle was defined as the incident angular range over which the optical efficiency remained above 90% of the maximum value, while the optical efficiency was defined as the ratio of the out-coupled optical power from the lightguide to the incident optical power on the lens array. Additionally, the ray-tracing parameters and optical conditions used in the simulations are summarized in Table 1.

**Table 1:** Ray-Tracing Parameters and Optical Conditions Used in the Simulations

Simulation parameter	Settings
Number of rays	2.5 × 10^6^ rays per simulation
Convergence criteria	Simulation terminated when the traced ray power fell below 1% of the source power.
Boundary conditions	Ambient environment was air, and no additional mounting fixtures were included in the optical model.
Source profile	Collimated source
Spectral weighting	AM1.5G solar spectrum, 300–1100 nm
Surface-scattering model	Fresnel equation considered for smooth optical surfaces
Angular distribution	Angular step: 1°; sweep range: –18° to +18°
Polarization	Unpolarized light assumed

Under ideal geometric assumptions, where V-groove reflectance and the transmittance of transparent surfaces were assumed to be unity, the model predicted an average optical efficiency of 88.3% over incident angles ranging from 0° to 15° at a wavelength of 550 nm. The optical efficiency is defined as the ratio of the out-coupled power from the LLA-SCPV system to the incident power on the system. Introducing Fresnel losses at interfaces reduced the optical efficiency to 79.2%. When the corner radius of the V-groove, *R* = 0.15 mm, was taken into account, the efficiency further decreased to 74.5%. Considering the measured PMMA transmittance and the AM1.5 spectral response within the solar cell absorption ranging from 300 to 1100 nm, the efficiency dropped to 58.1%. Accounting for the refractive index *n* and extinction coefficient *k* of the aluminum coating, as shown in Figure 3(a), the efficiency decreased to 50.3%. After accounting for the dead zone caused by the supporting structures and the inactive area of the solar cells, the final simulated efficiency was 47.9% on average for incident angles ranging between – 15° and 15°. The loss summary chart is shown in Figure 3(b).

**Figure 3:**
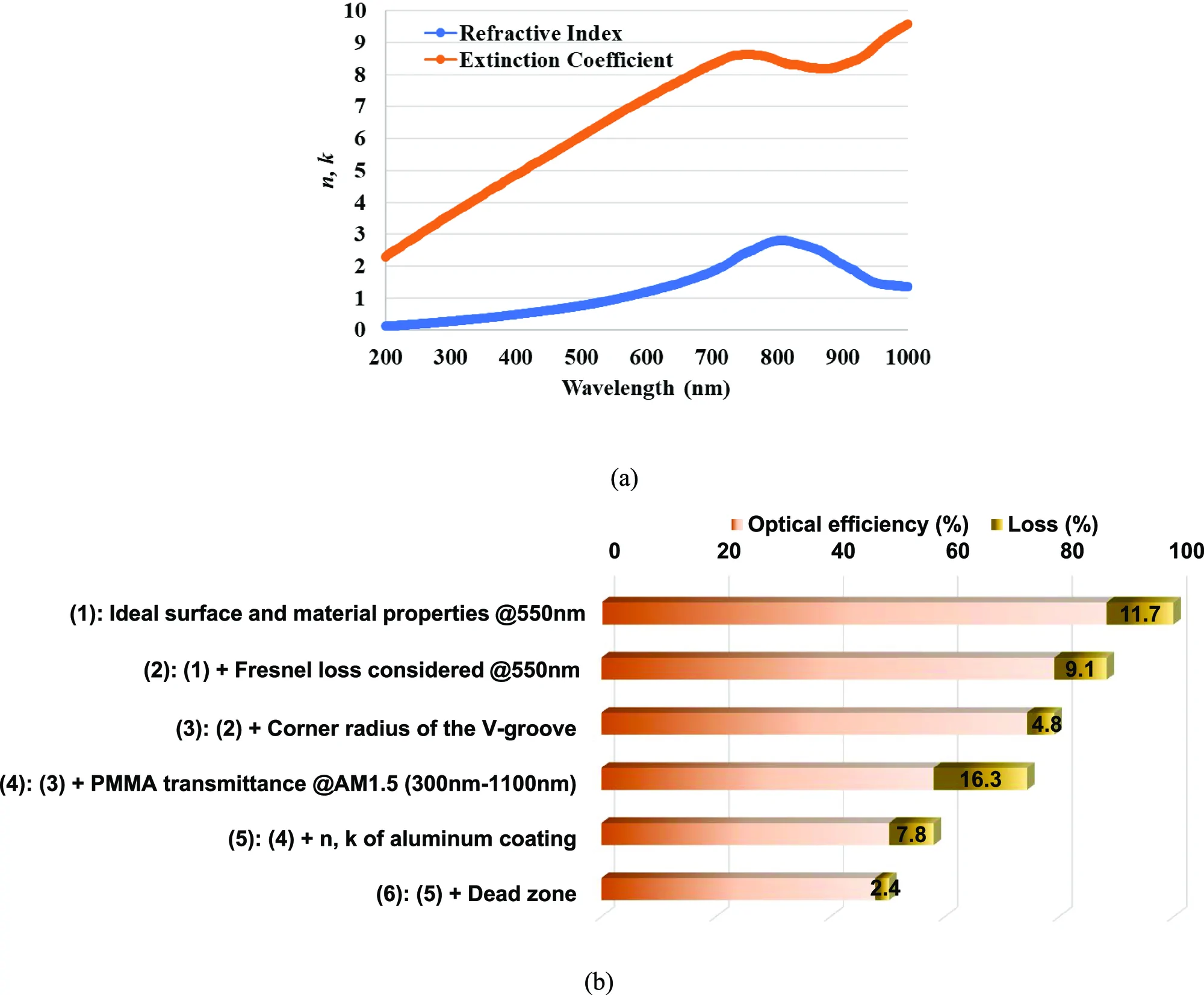
(a) The refractive index and the extinction coefficient of the aluminum coating used for system-performance prediction. (b) Simulated optical efficiency and the corresponding loss breakdown relative to the ideal case.

A fabrication and assembly tolerance analysis was conducted via simulation, as shown in Table 2. The model with the designed parameters was used as a baseline model, and its simulated optical efficiency was defined as the baseline efficiency, η_b_. The efficiency loss ratio *l*_r_ is defined as

1 $$l_{r}=\frac{\eta_{b}-\eta_{v}}{\eta_{b}}$$

where η_v_ is the simulated optical efficiency of a model with the varied parameter. The optical efficiency is highly sensitive to dimensional variations; even a 0.1% variation can result in considerable loss, indicating that a narrow tolerance range is required to minimize performance degradation.

**Table 2:** Fabrication and Assembly Tolerance Analysis

Parameter	Tolerance	Efficiency loss ratio
Base condition	-	0
Air gap	–0.1 mm ∼ +0.1 mm	–0.26% ∼ 0.48%
Solar-cell misalignment	–0.1 mm ∼ +0.1 mm	0.23% ∼ 0.08%
Dimension	–0.1% ∼ +0.1%	5.0% ∼ −4.8%
Adhesive thickness	–0.01 mm ∼ +0.01 mm	–0.02% ∼ 0.02%

## 3 Fabrication and Measurement

The profiles of the PMMA components in the proposed LLA-SCPV system were fabricated by a local third-party workshop using CNC machining. The lens array was manufactured as an aspheric plano-convex PMMA lens array. The PMMA lightguide was profiled with V-grooves whose facet angles followed the simulated design parameters. To provide the required reflectivity, the V-groove surfaces were coated with an aluminum layer after machining. The assembled module maintained an air gap of 2 mm between the lens array and the lightguide, consistent with the optimized design. The fabricated lens array, lightguide and the assembled prototype are shown in Figure 4(a)–(c), respectively.

**Figure 4:**
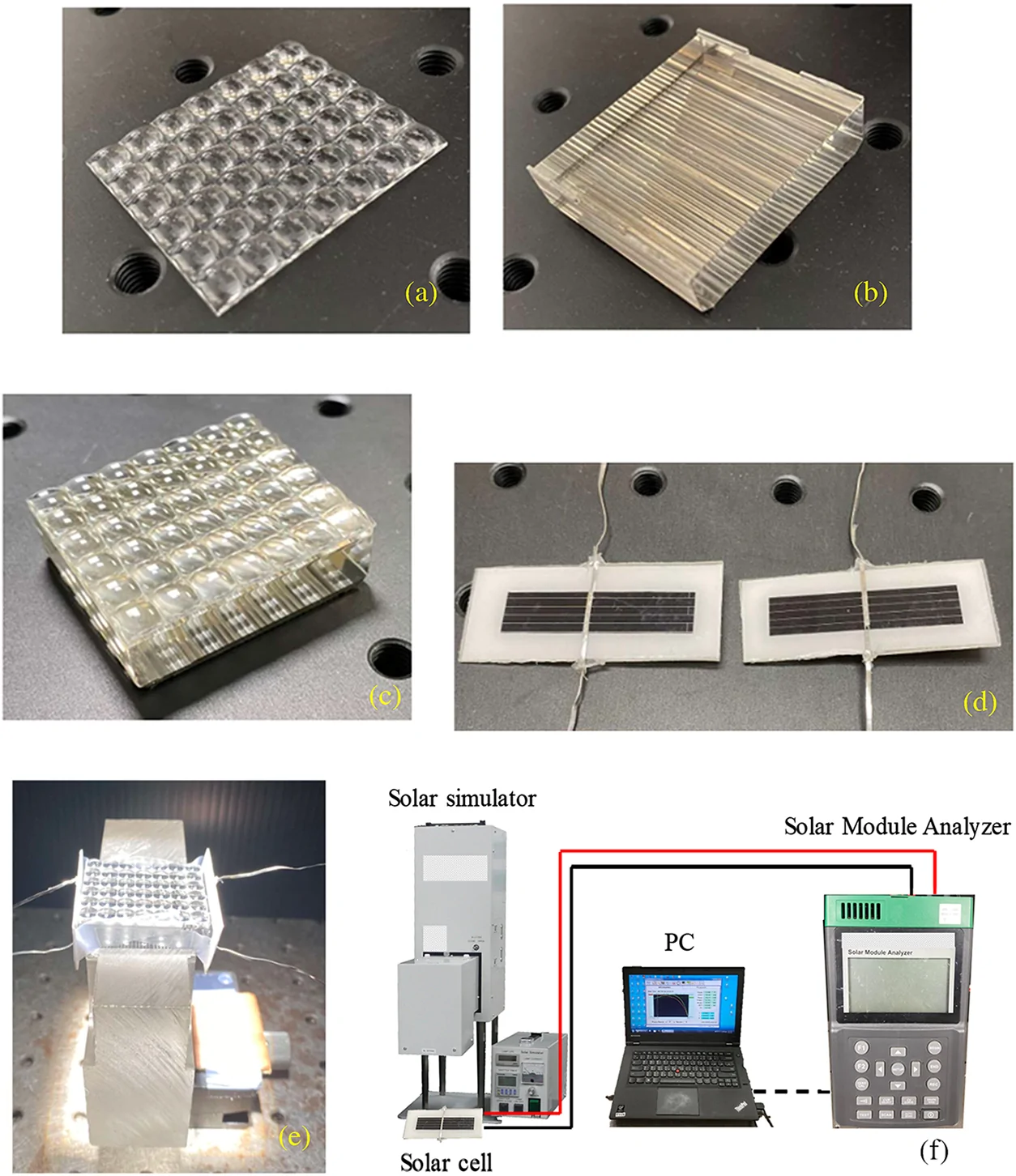
Prototype fabrication, assembly, and measurement. (a) CNC-machined PMMA lens array. (b) Lightguide after aluminum coating on the V-groove facets. (c) Assembled module showing the 2 mm air gap. (d) Customized solar cells. (e) Side-mounted solar cells and prototype mounted on a goniometer stage. (f) Measurement setup. Adapted with permission from ref 29

Experimental characterization was conducted using a solar simulator (Peccell PEC-L11) calibrated to reproduce the AM1.5G spectrum over an output wavelength range of 400–1100 nm. The simulator was used to evaluate the photovoltaic conversion efficiency of solar cells, as well as the optical efficiency and angular response of the fabricated prototype. The optical power of the light source was measured using a power meter (Ophir Nova II) equipped with a broadband sensor (Ophir 3A-P-FS-12, uncertainty ±1.9%). Prior to integration with the lightguide, the customized Si solar cells, as shown in Figure 4(d), were characterized under 1 sun illumination using the solar simulator to determine their photovoltaic conversion efficiency. The electrical outputs from the solar cells were recorded using a PV analyzer (PROVA 200A, uncertainty ±1%), and the measurement setup is illustrated in Figure 4(e). After this calibration step, two customized rectangular solar cells, each measuring 30 mm × 7 mm, were mounted on both sides of the lightguide to collect the out-coupled light, resulting in a geometrical concentration ratio of 2.5×. To investigate the angular performance of the LLA-SCPV prototype, the incident angle of the light source was adjusted using a goniometer stage (Thorlabs GNL10/M), as shown in Figure 4(f), and the optical alignment was verified prior to each measurement. Based on the measured solar cell conversion efficiency η_e_, and the measured output electrical power *P*_out,e_, the optical power delivered from lightguide to the solar cell was counted as

2 $$P_{\mathrm{out},\mathrm{opt}}=\frac{P_{\mathrm{out},e}}{\eta_{e}}$$

The incident optical power *P*_in,opt_ was calculated by the measured irradiance *I*, multiplied by the entrance area of the lens array, *A*. Therefore, the optical efficiency η_opt_ was determined as

3 $$\eta_{\mathrm{opt}}=\frac{P_{\mathrm{out},\mathrm{opt}}}{P_{\mathrm{in},\mathrm{opt}}}=\frac{P_{\mathrm{out},e}}{\eta_{e}\mathrm{IA}}$$

The measured output electrical power and the solar cell conversion efficiency were obtained by averaging the values measured from the left and right solar cells. The averaged output electrical power, the solar cell conversion efficiency, the irradiance, and the entrance area were *P*_out,e_ = (46.39 ± 0.46) mW, η_e_ = (19.9 ± 0.4) %, *I* = (1.00 ± 0.02) sun and *A* = 1050 mm^2^, respectively. The optical efficiency was calculated as (22.6 ± 0.5) %.

The acceptance-angle measurements were performed by rotating the prototype from −18° to +18° to cover the angular range used in the simulation. This setup enabled a direct comparison between simulated and measured optical performance.

## 4 Results and Discussion

The angular response of the LLA-SCPV system was evaluated through both ray-tracing simulations and experimental measurements. Figure 5 summarizes the angular dependence of optical efficiency under incident angles ranging from −18° to +18°, corresponding to the designed acceptance-angle window of the concentrator. In simulation, under a single wavelength of 550 nm and ideal geometric conditions, the optical efficiency remained above 82% from 0° to ±13° and above 78% at ±15°, demonstrating the combined effectiveness of the direct-aiming and TIR-assisted indirect-aiming mechanisms. As the incident angle increased beyond 13°, an efficiency drop was observed, primarily attributed to rays exceeding the TIR condition or missing the reflective V-groove facets, resulting in an acceptance angle of ±13° and an average optical efficiency of 88.3% within the incident angle of ±15°. When the Fresnel losses are further considered, a similar angular trend to that under ideal geometric conditions is observed, but with lower optical efficiency, resulting in an acceptance angle of ±13° and an average optical efficiency of 79.2% within ±15°. According to standard estimation conditions for solar cells and the specifications provided by component vendors, including the solar spectrum (AM1.5G), the absorption spectrum of Si solar cell (300–1100 nm), the round radius (*R* = 0.15 mm), the transmittance of the optical components, the reflectance spectrum of the V-groove facets (with an average reflectance of 69.8% within 300–1100 nm), and the dead zone of the solar cell, the simulated acceptance angle is ±12° and an average optical efficiency of 37.3% within ±15°. Experimental measurements exhibit a similar angular trend, confirming the broadened acceptance behavior enabled by the LLA-SCPV architecture. Specifically, the measured optical efficiency remained relatively stable within ±15°. A noticeable roll-off was observed as the incident angle exceeded this limit, a behavior that qualitatively aligns with the simulation despite the difference in the specific roll-off threshold. Nevertheless, the measured optical efficiency of the prototype was lower than the modeled values, mainly due to fabrication-induced imperfections. The corresponding analysis is presented and discussed in the following section.

**Figure 5:**
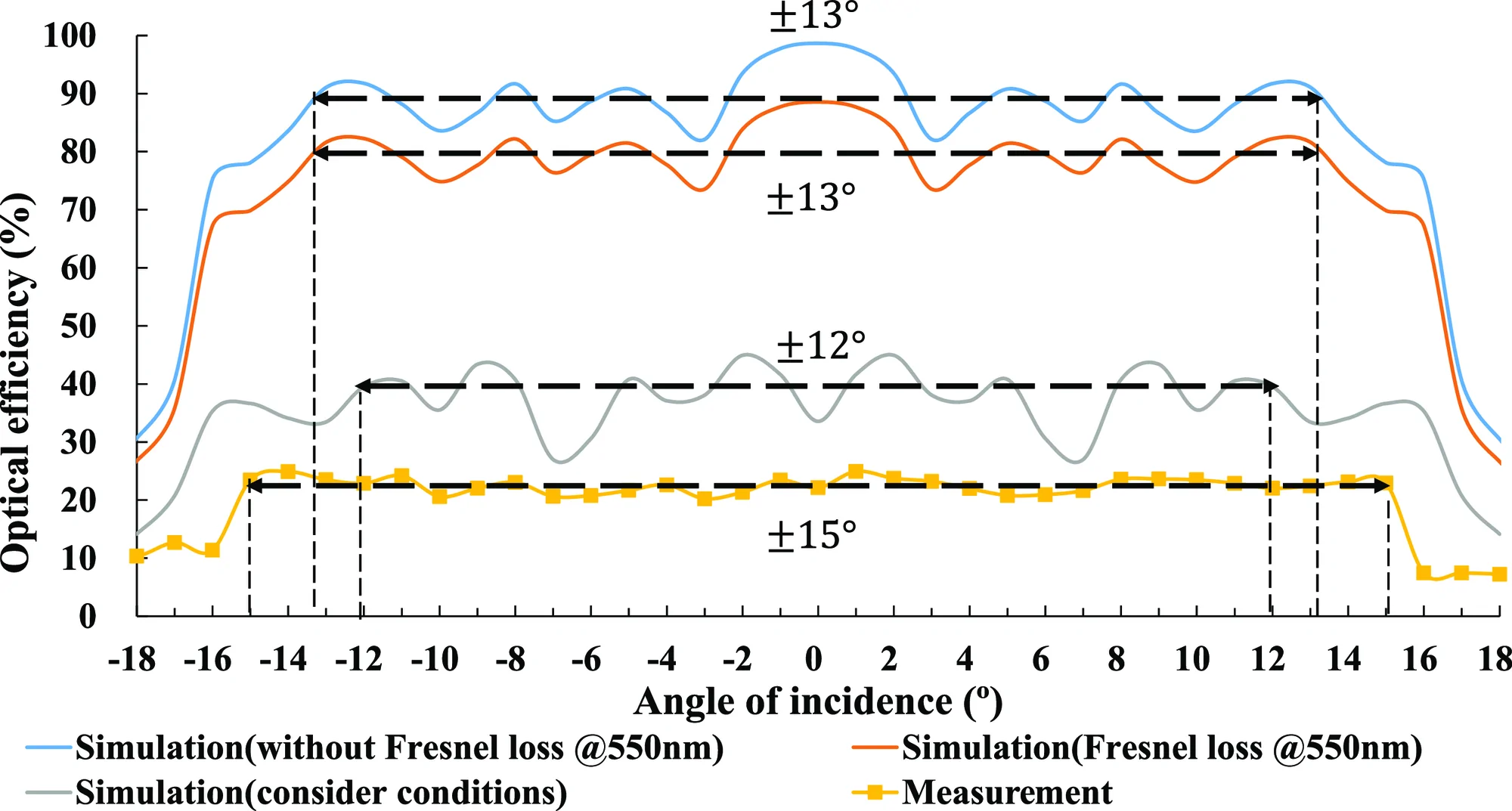
Angular responses of the LLA-SCPV, including simulated and measured results. Adapted with permission from ref 29.

According to the analyses via simulation, fabrication-induced imperfections can be categorized into two aspects: geometrical parameters and material properties. Geometrical deviations mainly arise from variations in the surface profiles of the optical components, particularly the V-groove facets. The surface profiles of the major V-groove facets were measured using a commercial profilometer (Dektak, Bruker) and compared with the design data, as shown in Figure 6(a). Meanwhile, the fabricated V-groove exhibits a blunted vertex instead of the designed sharp geometry, as shown in Figure 6(b). These deviations disrupted deterministic ray redirection toward the PV cells and increased optical loss, thereby reducing the average optical efficiency to 32.5% for incident angles within ±15°. The material-related issue arose from a discrepancy between the reflectance of the aluminum coating and the vendor specifications. The reflectance, measured using a commercial UV–vis spectrometer (OtO Photonics), is 45.8% on average over the wavelength range of 300–1100 nm, which is 24 percentage points lower than the specified value. This discrepancy lowers the average optical efficiency to 20.9% over the ±15° range of incidence. On the other hand, an index-matching optical adhesive was applied to connect the entrance surface of the solar cells with the out-coupling surfaces of the lightguide. Incorporating the adhesive into the simulation increases the optical efficiency of the proposed LLA-SCPV by 1.2 percentage points to 22.1%, in close agreement with the measured value of 22.6%. The aforementioned efficiency breakdown is illustrated in Figure 6(c).

**Figure 6:**
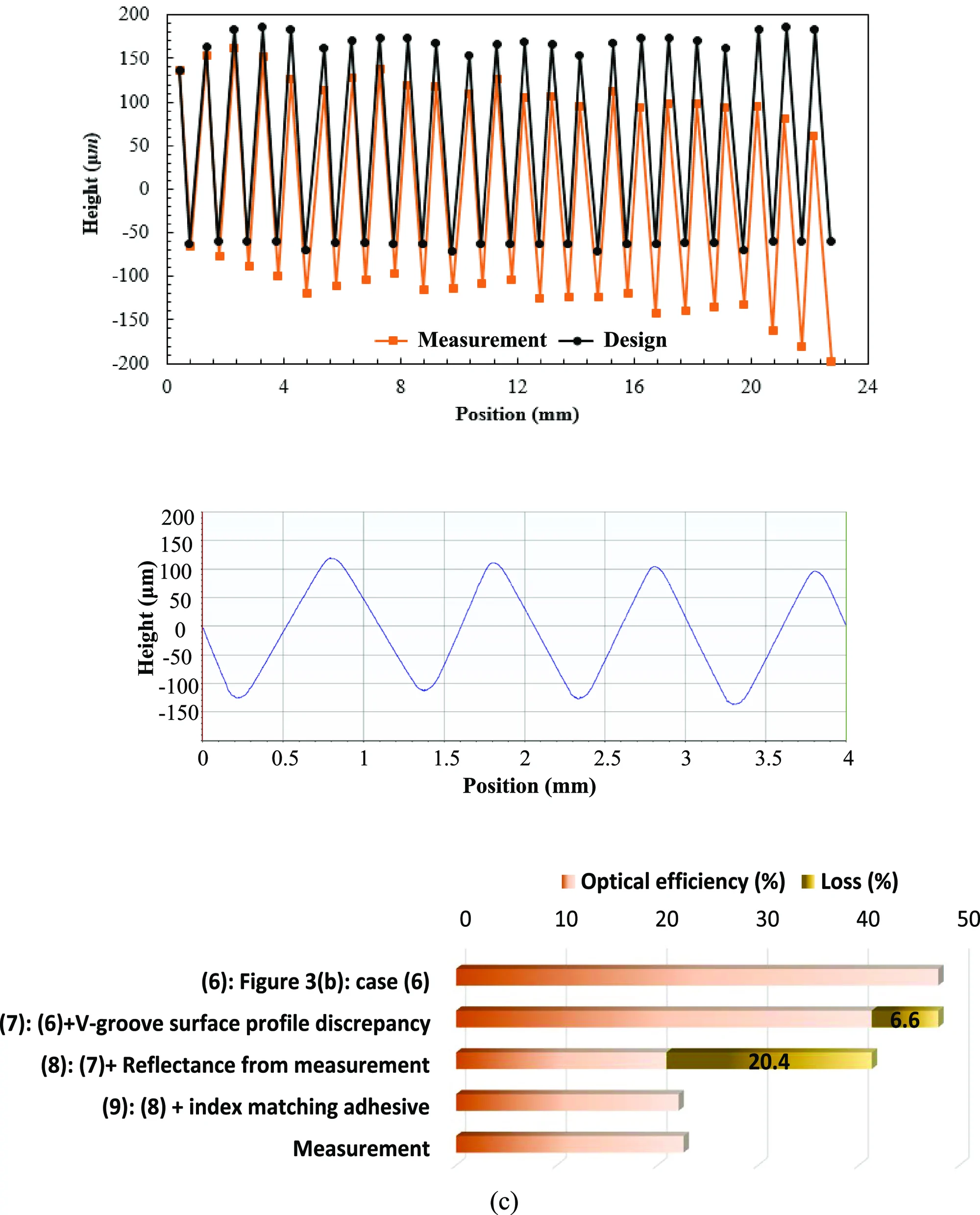
(a) Measured and designed profiles of the V-groove. Reprinted with permission from ref 29 (b) Enlargement of (a). Adapted with permission from ref 29 (c) Measured and simulated optical efficiency breakdown associated with fabrication-induced imperfections and index-matching optical adhesive.

The postanalysis of the angular response of the fabricated LLA-SCPV is illustrated in Figure 7. When the fabricated V-groove profile is incorporated into the simulation, the acceptance angle increases to approximately ±15.5°, showing good agreement with the experimental results. This indicates that fabrication-induced geometrical deviations are the primary factor responsible for the broadening of the angular response. According to the measured results shown in Figure 6(b), although the blunted vertex degrades the optical efficiency, its curved profile redistributes incident rays from deterministic paths and facilitates partial concentration, allowing rays outside the deterministic acceptance range to be partially redirected toward the PV cells and thereby broadening the angular acceptance. A comparison between simulation and measurement further reveals a flatter experimental response compared with the undulating simulated response. Given that the measured average surface roughness of the V-groove facets is *R*_a_ = 206 nm, surface roughness-induced scattering is expected to contribute to the observed flattened response.

**Figure 7:**
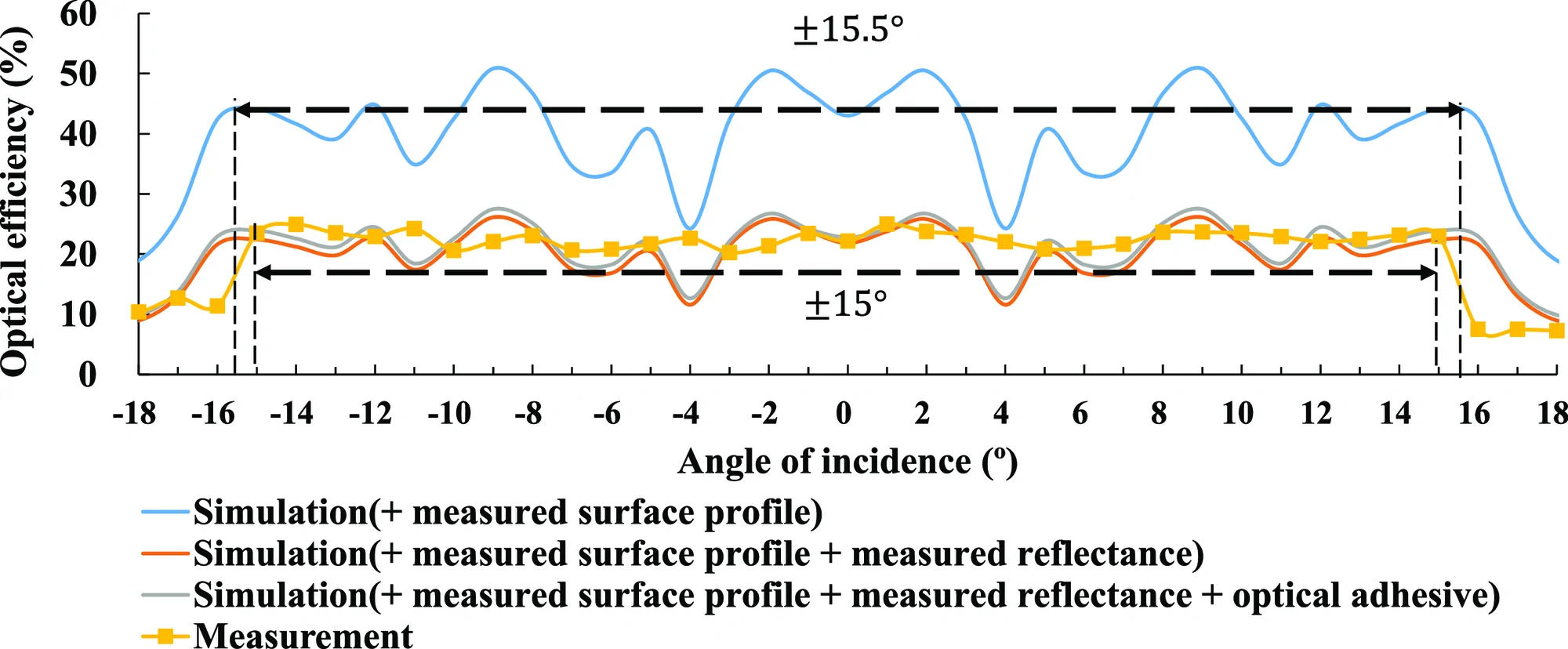
Postanalysis of the angular response. The simulation considers the measured V-groove surface profile and the coating reflectance, as well as the optical adhesive properties.

The reduced reflectance and the shape fidelity of the V-groove facets are identified as the dominant factors of the optical efficiency degradation. In this work, the lens array and lightguide were custom-fabricated by a third-party vendor through CNC machining and thermal evaporation. The reduced aluminum reflectance may be attributed to surface oxidation, surface roughness, and fluctuations in the coating process, such as temperature variations. Moreover, the nonplanar V-groove profile may have resulted in less uniform coating thickness and lower film quality than those typically achieved on flat substrates. These issues can potentially be mitigated through improved surface preparation, optimized deposition conditions, and improvement of the protective coating to enhance coating thickness uniformity, film quality, and oxidation resistance across the V-groove surfaces, thereby improving the reflectance of the aluminum coating. Using state-of-the-art fabrication technologies, the shape fidelity of the V-groove facets can also be improved by reducing the corner radius. Under realistic industrial capabilities, simulations indicate that replacing PMMA with fused silica via advanced techniques such as prevision glass molding, increasing the average reflectance of V-grooves to 0.86 across 300–1100 nm, and reducing the V-groove corner radius to 0.1 mm can substantially boost the optical efficiency to 67.7% and maintain the acceptance angle of approximately ±15.5°, providing a pathway for substantial performance enhancement.

For comparison, we categorized the aforementioned techniques into vertical-concentration and side-concentration groups, as shown in Table 3. The results shows that the proposed LLA-SCPV system achieves a wide acceptance angle of > ±12° among the side-concentration CPV systems. Particularly, compared with the previous design, in which the fabricated SCPV with V-grooves exhibited a maximum optical efficiency of 39.3% outdoors and an acceptance angle of ±4.4° in the indoor measurements, the present design achieved an average optical efficiency of 22.6% from the range of −15° to +15° and an acceptant angle of ±15.0°. In other words, the measured acceptance angle was improved by a factor of 3.4, with some sacrifice in optical efficiency. Therefore, the novelty of this work lies in the optical optimization of the lens-array/lightguide configuration to achieve wide-angle SCPV operation, offering the potential for wide-angle solar concentration while reducing tracking loads and tracker maintenance costs.

**Table 3:** Comparisons of CPV Techniques

System/Architecture	Acceptance angle	Optical efficiency	Concentration factor (C_g_)	Active area	Tracking requirement	Fabrication method	Measured output performance	refs
Vertical concentration	N/A	N/A	1–10,000× (design)	N/A	Required (UHCPV)	N/A	N/A (Simulation for solar cell)	^17^
Vertical concentration with bifacial solar cells	±62.6° (simulation)	84.3% (simulation)	3.83×	15 × 60 cm^2^	No tracking required	Reflective concentrator + bifacial PV	Experimental module efficiency: 8.3%–18.2% in a day	^20^
Vertical concentration with Si and III–V solar cells	±60° (experiment)	83.0% (simulation)	3.5× (III–V solar cell), 1× (Si solar cell)	4.05 × 4.47 cm^2^ (III–V solar cell), 32 × 40 cm^2^ (Si solar cell)	No tracking required	Silicone lens array and III–V/Si cells	Experimental module efficiency: 20.8%, simulated module efficiency: 27.3% when solar cells are improved.	^21^
Vertical concentration with CPC concentrator, Si and III–V solar cells	±62.3°, ±44.7°, ±35° (simulation for 3 designs)	78% (simulation)	2.25×, 4×, 6.25× (III–V solar cell, 3 designs), 1× (Si solar cell)	1 cm-diameter (III–V solar cell); 100 × 100 cm^2^ (Si cell)	No tracking required	N/A	N/A	^22^
Vertical concentration with tracking-integrated mechanism	±34°, ±33° (experiment for 2 designs)	80%–92% (experiment for 2 designs)	10×, 82.5× (2 designs)	5.5 × 5.5 mm^2^	Required (Tracking-integrated)	PMMA aspheric lens, III–V 3J PV and mechanical rails	Measured optical efficiency: ≥90% up to 20° (10× design), ≥80% up to 20° (82.5× design)	^23^
Vertical concentration	0.61° (simulation)	84.7% (simulation ideally) 25.8% (simulation with other issues), 23.1% (experiment)	4096× (simulation) 2400× (experiment)	5.5 × 5.5 mm^2^	Required	Fresnel lens and lightguide	Experimental optical efficiency: 23.1%	^19^
Vertical concentration/side-concentration for microscale CPVs (review)	Typical 0.5°–9.5°	65%–90% (on-axis data from measurement or simulation)	Typical 18.4×–1000×	54–1000 μm	Required (Various by design, such as tracking-integrated or low-cost external tracking)	Various micro-optical structures	Concentration ratio: Semprius Inc.: 1111×. Experimental module efficiency: Insolight S.A.: 36.4%, Semprius Inc.: 35%	^24^
Side-concentration CPV with V-groove	±4.4° (experiment)	85.5% (simulation) 39.3% (experiment)	5×	30 × 30 mm^2^	Reduced tracking requirement	PMMA lightguide with V-groove	Experimental acceptance angle: ±4.4°	^18^
Side-concentration CPV (ideal) Vertical concentration (demonstration)	±16°	37% (simulation, upscaling), 1.4% (experiment)	27× (simulation, upscaling)	1 × 53 mm^2^	Required (Passive self-tracking)	Phase-change-material self-tracking optics	Experimental effective concentration ratio: 3.5×	^25^
Side-concentration CPV microfluidic configuration	±25°	72% (experiment)	500× (simulation)	N/A	Required (Passive self-tracking)	Integrated microscale liquid optics for tracking	Experimental optical efficiency: 72%	^26^
Side-concentration CPV with lightguide-lens array	±12° – ±15.5° (simulation), ±15° (experiment)	22.6% (experiment)	2.5×	30 × 7 mm^2^	Reduced tracking requirement	PMMA lens array and V-groove lightguide	Experimental optical efficiency: 22.6%, acceptance angle: ±15°	This work

In summary, the proposed LLA-SCPV, featuring an extended acceptance angle can be employed to relax solar tracking requirements, thereby reduce tracking load and mechanical complexity. Furthermore, it can be deployed as modular tiles for opaque facade integration, representing a form of BIPV. Meanwhile, the LLA-SCPV is compatible with various photovoltaic technologies, particularly cost-intensive photovoltaics such as III–V solar cells, and those susceptible to UV-induced degradation, such as perovskite solar cells and perovskite-silicon tandem cells. Perovskite-based devices are known to be more vulnerable to heat and UV radiation than conventional inorganic solar cells. Accordingly, the proposed LLA-SCPV offers an alternative strategy for mitigating UV exposure by incorporating UV-filtering optical components.

## 5 Conclusions

In this study, a lightguide–lens–array side-absorption CPV (LLA-SCPV) was proposed and experimentally validated. The simulated acceptance angle of the proposed LLA-SCPV was found to be at least ±12°, while the experimentally measured acceptance angle was ±15°, demonstrating its potential for wide-angle CPV applications. This study also identifies and quantifies the root causes of optical efficiency loss. In particular, the lower-than-expected aluminum reflectance is identified as the primary factor governing efficiency degradation. Although fabrication-induced profile deviations in the V-groove facets result in significant optical efficiency loss, they also broaden the angular response. Meanwhile, surface roughness is responsible for the observed flattened angular response. Additionally, based on simulation results under realistic industrial capabilities, replacing PMMA with fused silica, increasing the V-groove reflectance to 0.86 (300–1100 nm) and reducing the corner radius to 0.1 mm can elevate the optical efficiency to 67.7% with an acceptance angle of approximately ±15.5°, highlighting a viable pathway toward substantial performance enhancement. Furthermore, the proposed LLA-SCPV further enables opaque facade integration and compatibility with cost-intensive photovoltaics and UV-sensitive perovskite-based photovoltaics, thereby facilitating integration into promising applications.

## References

[1] Best Research-Cell Efficiencies Chart. Rev. 03–19–2026; National Laboratory of the Rockies (NLR), 2026. (accessed April 2026).

[2] Vasilopoulou M., Huang D., Gong J., Li F., Zhang H., Sun Y. (2026). Tandem takeoff: Powering tomorrow with industrial-grade
perovskite/silicon solar cells. Adv. Energy
Mater..

[3] Basnet R., Yang Z., Ahmad V., Chang N., Fong K., Yan D. (2026). TOPCon-based
bottom cells for perovskite/silicon tandem
solar cells. Joule.

[4] Svarc, J. Most Efficient Solar Panels; Clean Energy Reviews, 2026. (accessed April 2026).

[5] Chen J., Zhou Q., Hu J., Li N., Steele J. A., Liu S. (2026). Revealing the Interface Degradation of Monolithic Perovskite/Silicon
Tandem Photovoltaics. ACS Energy Lett..

[6] Ahmad W., Kazmi J., Nawaz M. Z., Moutaouakil A. E., Zhang J., Illarionov Y., Wang Z. (2026). Interface engineering
in van der Waals heterostructures: enhancing photodetector efficiency
through structural and functional modifications. Adv. Funct. Mater..

[7] Ahmad W., Rehman M. U., Nawaz M. Z., Laxmi V., Huang J., Wu Y. (2025). Type-I band engineering of van der Waals InSe/TiS3
heterostructures for broadband and polarization-sensitive photodetection
with a large dichroic ratio. ACS Appl. Mater.
Interfaces.

[8] Ahmad W., Younis U., Nawaz M. Z., Laxmi V., Zhang J., Lei W. (2026). Interface engineering with graphene contacts in VP/MoS2
heterostructures for high-performance, durable, and multispectral
photodetection. ACS Appl. Mater. Interfaces.

[9] Wang H., Liu C., Xu R., Zhang Y., De Wolf S., Wang K. (2026). Upper interface
engineering between perovskite and electron transport layer toward
efficient and stable inverted perovskite solar cells. Adv. Mater..

[10] Wang T., Yi Z., Chen C. (2026). Interface engineering for high-performance perovskite
solar cells. Solar RRL.

[11] Fu Z., Xue M., Shao Z., Zhu Q. (2024). Performance evaluation of a novel
vacuum-tube PV/T system with inserted PV module and heat pipe. Renewable Energy.

[12] Mouaici K., Fersadou B., Arslan K. (2025). Technological
Limit
of Solar Concentration Technique Applied to Hybrid Photovoltaic-Thermal
Solar Collector Equipped with Thermoelectric Generator Incorporating
Ternary Nanofluid. ASME J. Sol. Energy Eng..

[13] Mariam E., Ramasubramanian B., Reddy V. S., Dalapati G. K., Ghosh S., Sherin T. (2024). Emerging trends in cooling technologies for photovoltaic
systems. Renewable Sustainable Energy Rev..

[14] Xie Y., You T., Dai B., Cui H. (2026). A review of colored building-integrated
photovoltaics (BIPV) for building decarbonization: Technological and
market perspectives. Renewable Sustainable Energy
Rev..

[15] Wang M., Mao P., Zhang P., Lv J., Po-Chuan Y., Li M. (2026). Lab-to-Fab: Advances
and Challenges in Bifacial Perovskite Solar
Cells and Modules. Adv. Funct. Mater..

[16] Gong J., Schneider L., Liu Y. (2026). Review on Perovskite Solar Cells:
From Single-Junction Devices to Tandem Deployment in Space. Adv. Sustainable Syst..

[17] Abushattal A. A., Loureiro A. G., Boukortt N. E. I. (2024). Ultra-high concentration vertical
homo-multijunction solar cells for cubesats and terrestrial applications. Micromachines.

[18] Pérez-Higueras P., Muñoz E., Almonacid G., Vidal P. G. (2011). High Concentrator
PhotoVoltaics efficiencies: Present status and forecast. Renewable Sustainable Energy Rev..

[19] Wei A. C., Hsiao S. Y., Sze J. R., Lee J. Y. (2019). V-groove and parabolic
array for enlarging the acceptance angle of a side-absorption concentrated
photovoltaic system. Opt. Laser Technol..

[20] Ceballos M. A., Fernández E. F., Rodrigo P. M., Valera Á., Pérez-Higueras P. J., Almonacid F. (2021). High-performance 4096× ultra-high CPV module based
on multiple concentrator units and optical guides. Opt. Lett..

[21] Shariah A., Hasan E. (2023). Design of a new static solar concentrator with a high concentration
ratio and a large acceptance angle based on bifacial solar cells. Clean Energy.

[22] Khalid M., Shanks K., Ghosh A., Tahir A., Sundaram S., Mallick T. K. (2021). Temperature regulation of concentrating photovoltaic
window using argon gas and polymer dispersed liquid crystal films. Renewable Energy.

[23] Sato D., Lee K. H., Araki K., Masuda T., Yamaguchi M., Yamada N. (2019). Design of low-concentration static
III-V/Si partial
CPV module with 27.3% annual efficiency for car-roof application. Prog. Photovoltaics: Res. Appl..

[24] Vu H., Vu N. H., Shin S. (2022). Static concentrator
photovoltaics
module for electric vehicle applications based on compound parabolic
concentrator. Energies.

[25] Ceballos M. A., Valera Á., Sanmartín P., Almonacid F., Fernández E. F. (2023). Development and indoor characterization
of a concentrator
photovoltaic assembly for tracking-integrated systems. Sol. Energy.

[26] Jost N., Gu T., Hu J., Domínguez C., Antón I. (2023). Integrated
micro-scale concentrating photovoltaic**s: a** scalable path
toward high-efficiency, low-cost solar power. Solar RRL.

[27] Zagolla V., Dominé D., Tremblay E., Moser C. (2014). Self-tracking solar
concentrator with an acceptance angle of 32°. Opt. Express.

[28] Kozodoy, P. ; Gladden, C. ; Pavilonis, M. ; Rhodes, C. ; Wheeler, T. ; Casper, C. Self-Tracking Concentrator for Photovoltaics; 2015 Conference on Lasers and Electro-Optics 2015, (CLEO 2015); IEEE pp 1–3 10.1364/CLEO_AT.2015.ATu2J.1.

[29] Hsu, Y. H. Improve acceptance angle of planar waveguide concentrator with solar system; Master’s Thesis; National Central University: Taiwan, R.O.C, 2023.

